# The ODHIN assessment tool: a tool to describe the available services for the management of hazardous and harmful alcohol consumption at the country and regional level

**DOI:** 10.1186/1940-0640-8-S1-A67

**Published:** 2013-09-04

**Authors:** Emanuele Scafato, Claudia Gandin, Miranda Laurant, Myrna Keurhorst, Marko Kolsek, Antoni Gual, Silvia Matrai, Jillian Reynolds, Joan Colom, Lidia Segura, Eileen Kaner, Dorothy Newbury Birch, Peter Anderson, Fredrik Spak, Preben Bendtsen, Hana Sovinova, Pierluigi Struzzo, Brzozka Krzysztof, Cristina Ribeiro, Van Schayck Onno, Gaby Ronda, Colin Drummond, Artur Mierzecki

**Affiliations:** 1 Istituto Superiore di Sanità (ISS), Italy; 2 Radboud University Nijmegen Medical Centre, the Netherlands; 3 Univerza V Ljubljani, Ljubljana, Slovenia; 4 Fundacio Privada Clinic per a la Recerca Biomedica /Hospital Clinico Provincial de Barcelona, Spain; 5 Department de Salut, Generalitat de Catalunya, Barcelona, Spain; 6 Newcastle University, Institute of Health and Society, UK; 7 University of Gothenburg, Sweden; 8 Linkoping University, Sweden; 9 Statni Zdravotni Ustav, Czech Republic; 10 Centro Regionale di Formazione per l’Area delle Cure Primarie, Udine, Italy; 11 Polish State Agency for Prevention of Alcohol-related Problems, Poland; 12 Istituto da Droga e da Toxicodependencia, Portugal; 13 Universiteit Maastricht, the Netherlands; 14 King’s College London, UK; 15 Pomeranian Medical University, Szczecin, Poland

## 

Optimizing Delivery of Health care Interventions (ODHIN) is an ongoing European project (EC, FP7) involving research institutions from 9 European countries using the implementation of Early Identification and Brief Intervention (EIBI) programmes for Hazardous and Harmful Alcohol Consumption (HHAC) in Primary Health Care (PHC) as a case study to better understand how to translate the results of clinical research into everyday practice. The Italian National Health Service (ISS) is the project leader of the Work Package 6 assessment tool. The aim of the ODHIN assessment tool is to formalise, operationalise and test the questionnaire developed under the PHEPA project in order to produce an update instrument to assess the extent of implementation of EIBIs for HHAC throughout PHC settings. The ODHIN assessment tool has been conceived as a semi-structured questionnaire for the identification of the state of the art, gaps and areas in the country that need further work and strengthening; to monitor the adequacy of brief intervention programmes for HHAC in order to provide recommendations to improve and optimize delivery of health care interventions. It analyses 24 questions distributed across 7 key sections. Data have been collected from 9 ODHIN collaborating countries (Catalonia, Czech Republic, Italy, Poland, Portugal, Slovenia, Sweden, The Netherlands and United Kingdom) and from other 14 European countries who have agreed to share their national experience with the ODHIN partners (Belgium, Croatia, Cyprus, Estonia, Finland, Fyrom-Yugoslav Republic of Macedonia, Germany, Greece, Iceland, Ireland, Latvia, Malta, Romania, and Switzerland). Preliminary data on the state of the art of the implementation and the extent of EIBI for HHAC throughout PHC settings across 23 European participating countries will be presented. Identified areas where services require development or strengthening across the participating countries as well as examples of good practices between countries will be also discussed.

